# The Treatment of Some Forms of Heart Disease

**Published:** 1892-12-31

**Authors:** 


### ROYAL FREE HOSPITAL.

### The Treatment of Some Forms of Heart Disease.

The treatment of heart disease should be governed more by symptoms than physical signs, for symptoms differ very little whatever the site of the disease.

There are many cases of marked structural lesion with no symptoms, because compensation is so far perfect; and treatment of these is directed to the preservation of the general health, as by tonics like syr. ferri. phosph., Easton's syrup, or small doses of arsenic, rather than directly to the heart, though Nature must be aided in her efforts to avert the ill-effects of the lesion by guarding against anything that may give additional labour to an already hard-worked heart. Such patients do not often come to our hospitals on account of heart disease, since till compensation begins to fail they do not feel much amiss; but, coming for some other complaint, the state of the heart is discovered. The patient himself should generally be kept in ignorance of the fact that there is anything wrong with his heart, but his friends are cautioned to keep him from violent exertion, over excitement, or mental worry; he is ordered to lead a regular and wholesome life, and to avoid any risk of chill.

Some cases come to the hospital complaining of cardiac pain, irregularity, and palpitation of the heart, dyspnœa, &c., and on examination the heart is found to be somewhat dilated, and there is a soft, mitral systolic murmur; the patient, who is anæmic, is gene rally in young life. The heart muscle is yielding fo- want of proper nutrition, and the following treatment would probably be prescribed:—

1. Avoid undue exertion, such as carrying heavy weights upstairs or walking fast uphill. In bad cases the patient would be confined to bed altogether for a time.

2. Take moderate exercise in the open air, being careful always to stop short of fatigue.

3. Avoid eating anything indigestible, but take plenty of simple, nutritious food.

4. In the way of drugs, if the state of the digestion will allow it, the patient is put upon iron, sometimes in the mild form of Blaud's pills, or citrate of iron and ammonia to begin with, and after this the tincture of the perchloride of iron, the bowels being carefully attended to. If the heart be irritable as well as weak, strychnia is very serviceable. Such patients are often quickly and permanently cured of their heart complaint, for the cause of their trouble—malnutrition—is removed.

These forms of heart disease have been considered first because their treatment—preventive in the first case and curative in the second—differs from that of most other forms, which must usually be only palliative. In most cases we cannot remove the cause, and so must be content to do the next best thing, and relieve the symptoms. Take such cases as those far from uncommon ones of mitral incompetence with hypertrophy and dilatation and secondary aortic mischief. It is impossible to cure these patients, but much can be done to palliate their sufferings and prolong their lives. Indeed, such cases are known to come into our hospitals time after time "to be patched up." In the worry and work of life compensation gives way, and the resulting dyspnœa and dropsy compel them to lie up and re-enter the ward, where the appropriate treatment sets them up again for a while; but each time they are admitted they seem worse than before, and the remedies less efficacious, till at last the heart is worn out and can be patched up no longer.

1. The patient is confined to bed, and kept as quiet as possible.

2. The diet is carefully attended to, for the patient needs nourishment, and yet the heart must not be taxed through the stomach. If they can be digested small meals of solid food are often found to be the best, because liquid distends the stomach and increases the bulk of the blood, thus embarrassing the heart and giving it more work to do. There is no routine diet for heart disease; the patient is given whatever it is found he can manage best.

3. He is carefully protected from cold and draughts, for the interference with the circulation through the lungs is sure to cause some passive congestion, and if bronchitis be set up by any exposure it is not unlikely to prove fatal. If cough is troublesome stimulating expectorants may be given, such as a mixture containing ammon. carb., tinct. scillæ, and sp. eth. co.

4. Cardiac patients often sleep very badly, and yet sleep is most important to them, and must be secured if possible. The effect of posture is a matter for consideration. Many mitral cases cannot lie down at all, but if they are carefully propped up and made comfortable with pillows they may sleep. Sometimes it is found necessary to give them a little opium or morphia.

5. The bowels are often constipated, but violent purgation is always avoided, for fear of faintness and collapse. The bowels are kept gently open by doses of the House Mixture or some mild aperient.

6. As regards drugs to act upon the heart itself, digitalis is found to be far and away the best cardiac tonic in most cases, not only of initial, but also of aortic disease, though in aortic incompetence it used to be thought not only useless but dangerous. It is specially indicated where there is a rapid, weak, and irregular action, for digitalis slows, strengthens, and steadies the heart. Like many drugs, its effects are cumulative, and people manifest idiosyncrascies with regard to it, so it is carefully watched, and stopped directly there is any sign of a poisonous effect. The indications looked for are (α) marked slowing of the pulse, which sometimes falls quite suddenly to as low as 40; (β) nausea or vomiting; and (γ) diminution in the amount of urine passed. No harm follows the administration of the drug if it be stopped on the appearance of any of these symptoms and the patient kept in the recumbent position for fear of syncope. Digitalis is generally prescribed in the form of the tincture; where it is not well borne or does not suit the patient strophanthus is sometimes substituted. Caffeine citrate is often prescribed in the wards, and is extremely useful for its action on the kidneys as well as on the heart. Alcohol is sometimes ordered, but it is generally reserved for use where there are sudden symptoms of failure and active stimulation is needed.

In chronic cases of heart disease, where the muscle is flabby, great advantage is found from the use of tonic doses of digitalis—about ten minims in twelve hours—combined with iron or strychnine, as in the following prescription: ℞. Vin. Ferri, ʒj; Tr. Dig. rg. v.; aq. ad., 3℥ gs., every four hours.

6. In the later stages of heart disease the patient is often greatly distressed by the accumulation of fluid in the great cavities and in the tissues of the body; the abdomen is distended, the fluid pressing upon the stomach and heart; serum collects in the pleural cavities, and further impedes the action of the congested and œdematous lungs; the legs and feet swell till the skin is tense and uncomfortable; the kidneys act very imperfectly, the urine being scanty and albuminous. Digitalis often removes the fluid in a marvellous manner, but it should be given in large and cumulative doses for a short time, and then stopped altogether or continued in diminished doses. Under its use as much as 200 ounces of urine has been passed in a day, and great relief been given to all the symptoms. The digitalis is often combined with the perchloride of iron, or caffeine citrate is sometimes substituted for it.

Tapping both of the pleural and abdominal cavities is occasionally performed for cardiac dropsy, but it is taught at the Royal Free that it is much better to remove the fluid by means of drugs, if possible. In one case of double mitral and aortic disease, with much œdema ascites, albumen in the urine, large liver, &c., the œdema was treated by Southey's tubes, one being inserted on the outer side, just about each ankle; but they were tiresome and would slip out at night, so instead of this the legs were propped up in a comfortable position, and a small incision made over the inner side of each ankle, the legs wrapped in boracic lint, and the fluid drained away for ten days; at the end of this time the legs were very much smaller. During the first twenty-four hours of this treatment 120 to 150 ounces of fluid were removed. The patient had been taking the following mixture: Tr. Ferri Perchlor., rg. x.; Tr. Scillae, rg. xx.; Pot Acetat, gr. xxv.; Sp. Ether. Nil., rg. xv.; Aq. ad. ʒj., every three or four hours. The effect of the treatment was to reduce the circumference of the abdomen from 38¼ to 31 inches, and the patient was soon able to be up and even assisting in the work of the ward.

In some cases of sudden failure of the heart, due to acute congestion of the lungs, where there is much cyanosis, it is advisable to have recourse to cupping or bleeding. In one such case of aortic disease 20 ounces of blood were removed, the patient recovered and lived for three months, dying then from another complaint.

